# The Chennai glaucoma study: Prevalence and risk factors for glaucoma in cataract operated eyes in urban Chennai

**DOI:** 10.4103/0301-4738.62655

**Published:** 2010

**Authors:** Ronnie George, Hemamalini Arvind, M Baskaran, S Ve Ramesh, Prema Raju, Lingam Vijaya

**Affiliations:** Medical and Vision Research Foundation, Sankara Nethralaya, Chennai, India

**Keywords:** Cataract surgery, glaucoma, population based study

## Abstract

We report the prevalence and risk factors for glaucoma among aphakes and pseudophakes in 3850 subjects who participated in a population-based study in urban south India. The subjects underwent an ophthalmic examination including applanation tonometry, gonioscopy, optic disc evaluation and frequency doubling perimetry. Glaucoma was diagnosed using the International Society of Geographical and Epidemiological Ophthalmology (ISGEO) criteria. Thirty eight, 15 aphakes and 23 pseudophakes (0.99% of 3850 subjects) of the 406 persons who had undergone cataract surgery were diagnosed with glaucoma. Aphakes/pseudophakes were at higher risk of glaucoma as compared to the phakic population (Odds Ratio: 2.71, 95% CI: 1. 94, 3.38, p=0.001). On multivariate analysis, older age and higher intra ocular pressure were risk factors for glaucoma. Blindness attributable to glaucoma was detected in 20% of aphakic and 4.3% of pseudophakic eyes. Glaucoma was a significant cause of morbidity in those who had undergone cataract surgery in this urban population.

Glaucoma is responsible for significant ocular morbidity in India. Primary glaucoma accounts for 2/3^rds^ of the morbidity.[1] A significant proportion of those who had undergone cataract surgery from the rural cohort of the Chennai glaucoma study had glaucoma.[2]

Since rural India is comparatively underserved in terms of both availability and the quality of ophthalmic care it is possible that the reported rate of aphakic and pseudophakic glaucoma is an exaggeration.[3] We report the prevalence and risk factors for glaucoma in aphakia and pseudophakia, among the urban population of the Chennai glaucoma study.

## Materials and Methods

The Chennai glaucoma study is a population-based study designed to report the prevalence of glaucoma in South India. The methodology has been described in detail elsewhere.[4] This study was approved by the Institutional Ethics Review Board and performed in accordance with the tenets of the Declaration of Helsinki for research; written informed consent was obtained from all participants.

All subjects underwent a comprehensive eye examination including slit lamp examination, Goldman applanation tonometry, gonioscopy, dilated fundus examination, optic disc examination using a 78 diopter (D) lens. Automated visual fields were performed for all the subjects with best corrected visual acuity of 4/16 or better, using frequency doubling perimetry (FDP; Carl Zeiss Meditec, Inc. Dublin, CA). All eligible subjects underwent C-20-1 screening, and the N-30 threshold test.[1]

A provisional diagnosis of suspected glaucoma was made if: intra ocular pressure (IOP) ≥ 21 mm Hg in either eye; vertical cup disc ratio (VCDR) ≥ 0.7 or a cup disc ratio (CDR) asymmetry ≥ 0.2; and focal thinning, notching, or a splinter hemorrhage. All these subjects were advised threshold visual field testing using the Swedish interactive threshold algorithm (SITA) standard 30-2 program (Humphrey Field Analyser model 750; Carl Zeiss Meditec).

Glaucoma was defined based on the International Society of Geographical and Epidemiologic Ophthalmology (ISGEO) classification.[5] Blindness was defined as a best corrected visual acuity of <2/40 (log MAR 1.3) and / or constriction of the visual field <10° from fixation, in the better eye.

Age, gender, IOP, peripheral anterior synechiae (PAS) ≥ 180°, pseudoexfoliation (PXF), diabetes,[6] hypertension[7] and duration from surgery were included to assess risk for glaucoma using both univariate and multivariate analysis (adjusted for age and gender). Significance was assessed at the *p*<0.05 level for all parameters and odds for glaucoma are presented with 95% CI's. Statistical analysis was carried out using SPSS 14.0 for Windows (SPSS Inc., Chicago, IL). All data is reported for patients and not eyes. Eyes with suspected primary glaucoma due to the presence of glaucoma in the phakic fellow eye or a diagnosis of glaucoma pre cataract surgery were excluded from the analysis.

## Results

Among the 4800 enumerated persons, 3850 (80.2%) participated. Of these, 2532 subjects performed normally and reliably, bilaterally, on supra threshold visual field testing using FDP. Normative limits based on these persons for VCDR and IOP for the population have been described earlier.[1]

Aphakia/pseudophakia was seen in 406 persons (10.54% of the entire population; age and gender adjusted prevalence 10.16%; 95% CI: 9.57-11.50%). Rural adjusted prevalence was 9.77% (95% CI: 8.84-10.69%). In the urban population, 49 (12.09%, 95% CI: 8.9, 15.24%) were diagnosed to have glaucoma, 11 were excluded from the analysis because they were considered to have primary glaucoma. Thirty eight of the 3850 persons examined (0.99%, 95%CI: 0.68, 1.30%) were diagnosed with glaucoma post cataract surgery. Those who had undergone surgery were significantly older (*p*<0.001) and more likely to have pseudoexfoliation (Odds Ratio (OR): 4.4, 95%CI: 2.2, 7.01) [Table 1]. Among the pseudophakes 23/318 (7.23%, 95% CI: 4.38, 10.07%) and 15/88 (17.05%, 95% CI: 9.19, 24.9%) aphakes were diagnosed to have glaucoma [Table 2].

**Table 1:** Comparison of phakic subjects with those post cataract surgery

	Phakic(3444)	Aphakia/Pseudophakia(406)	*‘P’* value	Odds Ratio(95%CI)
Mean age (SD)years	53.55 (9.79)	66.06 (9.42)	0.0001	
Females (percentage)	1931 (56.1%)	209 (51.5%)	0.08	0.83 (0.68,1.21)
IOP (mm Hg)(SD)	16.35 (4.25)	16.59(6.11)	0.31	
Pseudoexfoliation (percentage)	57 (1.66%)	28 (6.9%)	0.0001	4.4 (2.77,7.01)
Blindness secondary to glaucoma(unilateral)(percentage)	12(0.35)	3(0.74)	0.43	2.13 (0.60,7.58)

SD – Standard deviation, IOP – intraocular pressure

**Table 2:** Demographic and ocular variables for subjects with post cataract surgery glaucoma compared to those who had undergone cataract surgery

	No glaucoma (368)	Glaucoma (38)	*‘P’* value	Odds Ratio (95%CI)
Mean age (SD) years	65.7 (9.5)	69.7 (7.6)	0.01	
Aphakes (percentage)	68.0 (10.1)	71.3 (5.3)	0.47	
Pseudophakes (percentage)	64.9 (9.2)	68.6 (8.7)	0.65	
Sex (M:F)	179:189	18:20		1.05(0.54,2.05)
Aphakes (M:F)	37:36	6:9	0.58	0.64 (0.20,2.01)
Pseudophakes (M:F)	142:153	12:11	0.82	1.17 (0.50,2.74)
IOP (mm Hg)(SD)	16.1(5.1)	21.9 (12.3)	<0.001	
Aphakes (mm Hg) (SD)	16.2 (5.6)	23.5 (12.7)	0.01	
Pseudophakes (mm Hg) (SD)	16.0 (4.9)	20.9 (12.3)	<0.001	
Pseudoexfoliation (n)	23	5	0.58	2.27 (0.81,6.37)
Aphakes	9	2	0.65	1.14 (0.22.5.92)
Pseudophakes	14	3	0.13	3.01 (0.8,11.35)
PAS≥ 180 degrees (n)	6	2	0.94	3.37 (0.66,17.32)
Aphakes	4	0	1	2.00 (0.1,39.28)*
Pseudophakes	2	2	0.005	3.38 (0.45,25.58)
Mean Sx-Ex (SD) months†	58.4 (51.60)	79.4 (71.5)	0.03	
Aphakes(months)	102.5 (64.8)	126.8 (87.1)	0.24	
Pseudophakes (months)	41.2 (31.9)	50.0 (39.0)	0.24	

* 0.5 was added to all cells for calculation,

† Mean surgery to examination duration, SD – Standard deviation, IOP – intraocular pressure, PAS – peripheral anterior synechiae

Persons who had undergone cataract surgery were at higher risk of glaucoma as compared to the phakic population (OR: 2.71, 95%CI: 1.94, 3.38, p=0.001). On univariate analysis, significant risk factors for glaucoma included older age, higher IOP, aphakia and longer duration from surgery [Table 2]. On multivariate analysis age (OR: 1.07; 95% CI:1.02, 1.12, p=0.003), increasing IOP (1.13; 95% CI:1.06,1.21, *p*<0.001,) and PAS ≥ 180° (OR: 7.7, 95% CI:1.1,54.4, p=0.04) were associated with increased risk of disease.

Twenty percent of aphakes (3/15) and 4.3% of pseudophakes (1/23) were unilaterally blind due to glaucoma. Aphakia was associated with higher risk of blindness due to glaucoma (OR: 11.15, 95%CI: 1. 2, 108.9, p=0.03) as compared to pseudophakia. None of the subjects were bilaterally blind [Table 3].

**Table 3:** Comparison of demographic and ocular variables for subjects with aphakia and pseudophakia

	Pseudophakia (318)	Aphakia (88)	*‘P’* value	Odds Ratio (95%CI)
Mean age (SD) years	65.5 (9.2)	68.0 (9.9)	0.02	
Sex (M:F)	154:164	43:45	0.94	1.01 (0.63,1.63)	
IOP (mm Hg (SD))	16.3 (5.7)	17.8 (7.2)	0.04	
PEX (%age)	17 (5.3%)	11 (12.5%)	0.04	2.52 (1.14,5.62)
PAS≥180 degrees (%age)	4 (1.3%)	4 (4.5%)	0.07	3.73 (0.92,15.63)
Mean Sx-Ex (SD) months *	43.4 (33.4)	117.1 (69.5)	<0.001	
Glaucoma (%age)	23 (7.23%)	15 (17.05%)	0.009	2.64 (1.31,5.30)
Blindness (%age) (unilateral)	1 (0.3)	3 (3.4)	0.03	11.15 (1.15,108.9)

* Mean surgery to examination duration, SD – Standard deviation, IOP – intraocular pressure, PEX - pseudoexfoliation, PAS – peripheral anterior synechiae

## Discussion

Glaucoma was diagnosed in 9.36% (95% CI: 6.5, 12.2%) of aphakes/pseudophakes in this urban cohort. These numbers are similar to those reported for the rural cohort (11.2%, 95%CI: 8.38, 14.01).[2] This is higher than that reported in most other studies.[8,9] except the Zulu population among whom the rates of aphakic glaucoma were higher (33%)[8]

From an etiologic perspective, glaucoma in aphakes/pseudophakes could be either - pre-existing, developed de novo or secondary to surgical trauma. We have tried to exclude primary glaucoma by excluding those with glaucoma in the phakic fellow eye. Preexisting glaucoma could remain undiagnosed if an inadequate preoperative evaluation was performed. Thirty nine per cent of the phakic subjects with glaucoma in this urban population had significant cataract. If the healthcare system could detect these cases when they present for cataract surgery it would dramatically improve the detection rates for glaucoma in the population (current detection rates are 7.8% for primary glaucoma in this urban cohort[1] and 1% for the rural sample).

From our results it appears that the availability of better eye care facilities in an urban population does not necessarily translate into lower rates of post cataract surgery glaucoma, even though the visual outcomes of surgery in the urban cohort were better than in the rural cohort.[10] For many people in the country the only point of contact with the eye care system is when they seek or are “screened” for cataract surgery. Inadequate or inappropriate examination at this time is a lost opportunity to detect and treat other non cataract ocular pathology.

Our findings that nearly 10% of the population aged 40 years and older, who had undergone cataract surgery, had glaucoma, highlight the need for a comprehensive evaluation pre cataract surgery and improved quality of cataract surgery. In addition, with increasing life expectancy those undergoing cataract surgery would be at risk of developing glaucoma with time and should be encouraged to continue to undergo periodic eye examinations.
